# Movement of Lipid Droplets in the Arabidopsis Pollen Tube Is Dependent on the Actomyosin System

**DOI:** 10.3390/plants12132489

**Published:** 2023-06-29

**Authors:** Lang Yang, Jinhong Liu, Ching-Kiu Wong, Boon Leong Lim

**Affiliations:** 1 School of Biological Sciences, University of Hong Kong, Pokfulam, Hong Kong, China; u3005772@connect.hku.hk (L.Y.); u3006988@connect.hku.hk (J.L.); ester113@connect.hku.hk (C.-K.W.); 2 State Key Laboratory of Agrobiotechnology, The Chinese University of Hong Kong, Hong Kong, China

**Keywords:** *Arabidopsis thaliana*, pollen tube, lipid droplets, myosin, actin

## Abstract

The growth of pollen tubes, which depends on actin filaments, is pivotal for plant reproduction. Pharmacological experiments showed that while oryzalin and brefeldin A treatments had no significant effect on the lipid droplets (LDs) trafficking, while 2,3-butanedione monoxime (BDM), latrunculin B, SMIFH2, and cytochalasin D treatments slowed down LDs trafficking, in such a manner that only residual wobbling was observed, suggesting that trafficking of LDs in pollen tube is related to F-actin. While the trafficking of LDs in the wild-type pollen tubes and in *myo11-2*, *myo11b1-1*, *myo11c1-1*, and *myo11c2-1* single mutants and *myo11a1-1*/*myo11a2-1* double mutant were normal, their trafficking slowed down in a myosin-XI double knockout (*myo11c1-1*/*myo11c2-1*) mutant. These observations suggest that Myo11C1 and Myo11C2 motors are involved in LDs movement in pollen tubes, and they share functional redundancy. Hence, LDs movement in Arabidopsis pollen tubes relies on the actomyosin system.

## 1. Introduction

The pollen tube, the fastest-growing cell, is fundamental to the reproductive success of all flowering plants. After landing on the stigma of a flower, the pollen grain germinates to generate a pollen tube, which penetrates the style to deliver sperm cells to the embryo. Pollen tube growth is a polarized growth process involving the functional coordination of various organelles. Cytoplasmic streaming of vesicles and organelles and reorganization of the cytoskeleton occur rapidly during pollen tube growth [1,2]. During pollen maturation, pollen accumulates lipid droplets (LDs) as a carbon and energy source for subsequent pollen germination [3,4]. LDs have also been called lipid bodies, oil bodies, spherosomes, and oleosomes [5,6,7,8], which are derived from the endoplasmic reticulum (ER) and are present in most eukaryotic cells [9,10]. Previously, LDs were thought to be particles in cells that store or release energy, but it was not until the last decades that LDs were recognized as an indispensable organelle, which has special lipid and protein compositions and the unique topology of a half-unit membrane [11,12]. LDs are mainly composed of triglycerides (TAG) and are encircled by a phospholipid monolayer and play pivotal roles in lipid and energy homeostasis [13,14]. LDs biogenesis in pollen involves the de novo biosynthesis of fatty acids in plastids producing acyl-CoA, which is added to glycerol-3-phosphate (G3P) to generate TAG [15,16]. 

The accumulation of LDs during pollen maturation is critical to pollen viability, pollen germination, and successful reproduction [17]. Mobilization and the breakdown of LDs stored in the pollen grains are also important for the initiation of pollen germination [18,19]. In addition to its role as an initial source of energy and carbon sources at the beginning of pollen germination, the breakdown of triacylglycerols (TAGs) in LDs is also suggested to release fatty acids for the synthesis of the plasma membrane of the pollen tube [20]. While starch and lipid reserves in pollen grains provide the carbon skeletons and energy necessary for the initial stages of pollen germination, a continuous supply of sucrose from the style is required to sustain pollen tube growth [21]. Exogenous sucrose serves as a carbon and energy source for fatty acids and lipid synthesis during pollen tube growth [22,23]. As a result, new LDs are synthesized during pollen tube growth [21]. 

In *Arabidopsis thaliana* (Arabidopsis), the transport of the mitochondria, peroxisomes, ER, Golgi apparatus, and LDs are dependent on the actomyosin system [11,24,25,26,27]. A pollen tube is a single cell and its growth is dependent on actin filaments. Myosin is an actin-based motor protein that transmits energy from ATP hydrolysis into the movement of actin filaments. Myosins are classified into at least 79 different classes through all eukaryotes based on sequence similarity [28]. In plants, only class VIII and XI myosins are present. Arabidopsis myosins fall into 4 class VIII genes and 13 class XI genes [29]. Seven of the thirteen myosin XI genes are expressed in pollen: XI-2, XI-A (Myo11A1), XI-B (Myo11B1), XI-C (Myo11C1), XI-D (Myo11A2), XI-E (Myo11C2), and XI-J [30,31]. Inactivation of myosin XI-2 or XI-K resulted in defects in root hair elongation [32]. In Arabidopsis, single mutants of the seven myosin XI genes expressed in pollen had no obvious changes in fertility, whereas *myo11c1-1*/*myo11c2-1* double mutant had defects in pollen tube growth, slower movements of the Golgi stack, and peroxisomes and showed less-organized actin filaments in the pollen tube, suggesting that myosin XI Myo11C1 and Myo11C2 play critical roles in pollen tube growth, organelle motility, and actin organization.

However, the mechanisms of the movement of LDs in pollen tubes remained largely unknown. In this study, we investigated the movement of LDs in WT and several myosin XI mutants in pollen tubes. We also examined the effects of 2,3-butanedione monoxime (BDM), oryzalin, brefeldin A, cytochalasin D, SMIFH2, and latrunculin B treatments on LDs movement in pollen tubes. Pharmacological experiments indicated that relative to the untreated control, LDs movement was affected by BDM, cytochalasin D, SMIFH2, and latrunculin B treatments, whereas brefeldin A and oryzalin treatments did not significantly reduce the mobility of LDs in pollen tubes. Relative to WT pollen tubes, the LDs movement in *myo11-2*, *myo11a1-1*/*myo11a2-1*, *myo11b1-1*, *myo11c1-1*, and *myo11c2-1* mutants pollen tubes were not altered significantly. However, LDs movement in the *myo11c1-1*/*myo11c2-1* double mutant was moderately slower, suggesting that the movement of LDs in pollen tubes is mainly dependent on F-actin and myosin XI Myo11C1 and Myo11C2.

## 2. Results

### 2.1. Tracking LDs Movement in Pollen Tubes in Arabidopsis thaliana

To examine if the actomyosin system is involved in LDs’ movement, we conducted pharmacological experiments to examine if the mobility of LDs in pollen tube was affected by the myosin ATPase inhibitor, 2,3-butanedione monoxime (BDM) [33]; the vesicle formation drug, brefeldin A [34]; the microtubule depolymerizing drug, oryzalin [35]; F-actin polymerization inhibitors, SMIFH2 [36]; F-actin depolymerization drug, latrunculin B and cytochalasin D [11,37]. 

**Figure 1:**
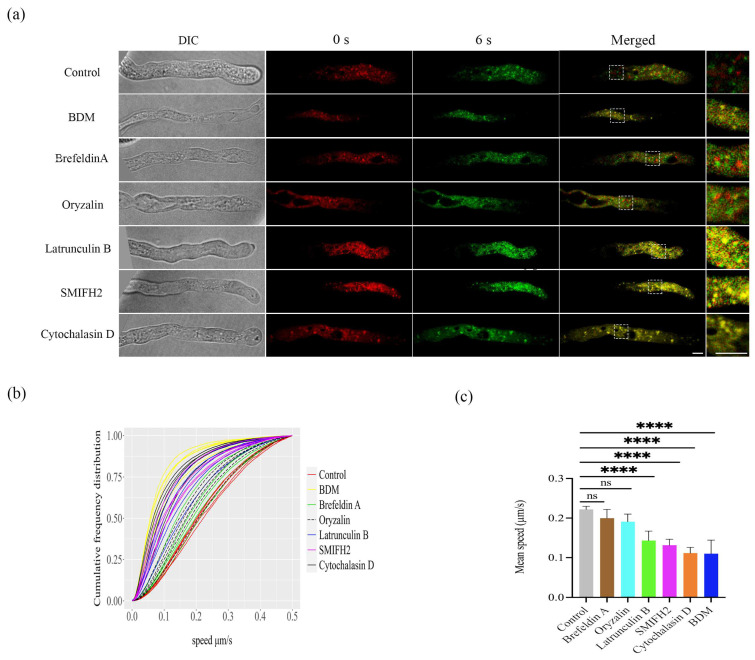
Effects of different pharmacological agents on the mobility of LDs in WT pollen tubes. (**a**) LDs’ movement was imaged at 0 s (red signals) and 6 s (green signals). Some pollen tubes were treated with 10 µM oryzalin, 50 µM brefeldin A, 40 µM cytochalasin D, 100 µM SMIFH2, 3 nM latrunculin B, and 50 mM 2,3-butanedione monoxime (BDM), respectively. LDs in pollen tubes were stained by the neutral lipid stain Nile Red. The 0 s DIC (differential interference contrast) image was chosen as the representative one. 3× enlarged merged images were shown at the right. Scale bar = 2 µm. (**b**) The cumulative frequency distribution curve of the speed of pollen tubes LDs in 5 min time-lapse images. (**c**) The mean velocity of lipid droplets movements in pollen tubes treated with 10 µM oryzalin, 50 µM brefeldin A, 40 µM cytochalasin D, 100 µM SMIFH2, 3 nM latrunculin B, and 50 mM 2,3-butanedione monoxime (BDM), for 15 min were compared with that of untreated pollen tubes. The results are expressed as the mean ± standard error; *n* = 5. “ns” indicates no significant differences between control and treatments. Asterisks indicate significant differences between control and each of BDM, latrunculin B, SMIFH2, and cytochalasin D treatments (**** *p* < 0.0001).

Hardly any co-localization of fluorescence signals between LDs from 0 s with 6 s was observed in the control group, or pollen tubes treated with brefeldin A and oryzalin (Figure 1a). However, fluorescence signals of LDs of 0 s overlapped with LDs’ signals of 6 s in pollen tubes treated with BDM, latrunculin B, SMIFH2, and cytochalasin D (Figure 1a), suggesting that these inhibitors slowed down LDs’ movement in the pollen tubes. LDs’ velocities were measured for each frame by an automated algorithm. Instantaneous speeds for each LD in every pollen tube were plotted as cumulative frequency distribution graphs (Figure 1b). Lipid droplets moved more slowly in pollen tubes treated with BDM (yellow lines), latrunculin B (blue lines), SMIFH2 (pink lines), and cytochalasin D (black lines) than untreated pollen tubes (red lines) (Figure 1b). The average speed of LDs in pollen tubes was 0.22 ± 0.01 µm s^−1^ (Figure 1). Compared to the controls (Figure 1c; Video S1), the trafficking of LDs under brefeldin A (0.20 ± 0.02 µm s^−1^) (Video S2), and oryzalin (0.19 ± 0.02 µm s^−1^) (Video S3) treatments were very similar and showed no significant differences. Nevertheless, the mobility of LDs in the pollen tubes was significantly reduced by BDM (0.09 ± 0.02 µm s^−1^), latrunculin B (0.14 ± 0.02 µm s^−1^), SMIFH2 (0.13 ± 0.01 µm s^−1^), and cytochalasin D (0.11 µm s^−1^ ± 0.01) treatments (Figure 1). BDM (Video S4), Latrunculin B (Video S5), SMIFH2 (Video S6), and cytochalasin D (Video S7) treatments restricted LDs’ trafficking, and only residual wobbling was observed. In summary, these results indicate that LDs’ trafficking requires F-actin, but not microtubules.

### 2.2. Mean Speed of the LDs in Pollen Tubes in WT and Myosin Mutants

Pharmacological experiments showed that LDs’ trafficking in pollen tubes requires F-actin. The movement of mitochondria in cultured tobacco cells was dependent on F-actin and myosin ATPase as the mitochondrial movement in the culture tobacco cells was inhibited by the myosin ATP inhibitor BDM [33]. Herein, Arabidopsis pollen tubes, the myosin ATPase inhibitor BDM also inhibited LDs’ movement (Figure 1). Therefore, we further examine if the LDs’ movement is normal in pollen-specific myosin XI mutants (Table S1). 

**Figure 2:**
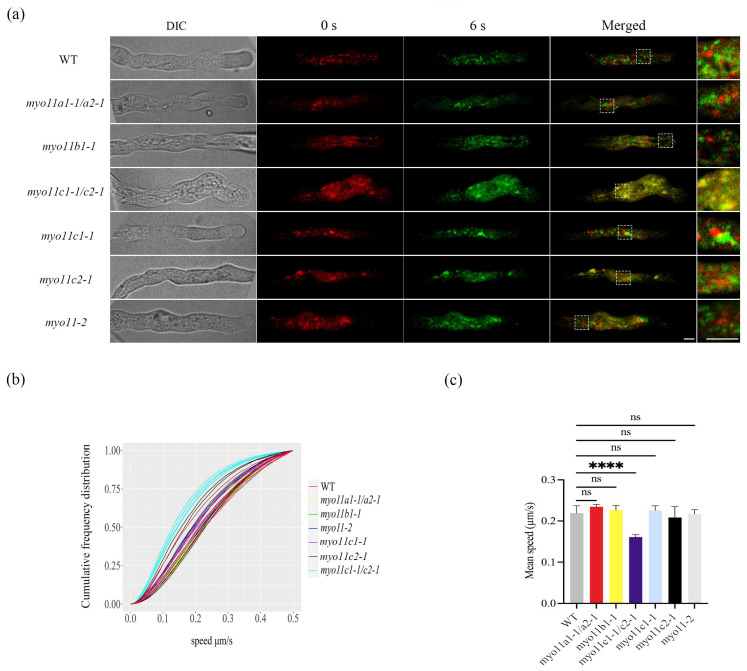
Movement of LDs in the pollen tubes of myosin mutants and WT plants. (**a**) LDs’ movement was imaged at 0 s (red signals) and 6 s (green signals) in pollen tubes of WT and *myo11a1-1*/*myo11a2-1*, *myo11b1-1*, *myo11c1-1*/*myo11c2-1*, *myo11-2*, *myo11c1-1*, and *myo11c2-1* mutants. LDs in pollen tubes were stained by the neutral lipid stain Nile Red. The 0 s DIC (differential interference contrast) image was shown at the left. 3x enlarged merged images were shown at the right. Scale bar = 2 µm. (**b**) The cumulative frequency distribution curve of the speed of LDs imaged in 5 min time-lapse in the pollen tubes of WT and *myo11a1-1*/*myo11a2-1*, *myo11b1-1*, *myo11c1-1*/*myo11c2-1*, *myo11-2*, *myo11c1-1*, and *myo11c2-1* mutants. LDs in *myo11c1-1*/*myo11c2-1* pollen tubes (blue lines) moved more slowly than in WT pollen tubes (red lines). (**c**) The mean velocity of LDs’ movements in pollen tubes in WT and *myo11a1-1*/*myo11a2-1*, *myo11b1-1*, *myo11c1-1*/*myo11c2-1*, *myo11-2*, *myo11c1-1*, and *myo11c2-1* mutants was analyzed. The results are expressed as the mean ± standard error; n = 5. “ns” indicates no significant differences between WT and each of the *myo11a1-1*/*myo11a2-1*, *myo11b1-1*, *myo11-2*, *myo11c1-1*, and *myo11c2-1* mutants. Asterisks indicate significant differences between WT and *myo11c1-1*/*myo11c2-1* (**** *p* < 0.0001).

Very little overlap between fluorescence signals of lipid droplets collected at 0 s and at 6 s was detected in the pollen tubes of wild type, *myo11a1-1*/*myo11a2-1*, *myo11b1-1*, *myo11-2*, *myo11c1-1*, and *myo11c2-1* mutants (Figure 2a). In sharp contrast, fluorescence signals of lipid droplets at 0 s almost overlapped with lipid droplet signals at 6 s in the pollen tube of *myo11c1-1*/*myo11c2-1* double mutant (Figure 2a), suggesting that the LDs’ movement was slower in the pollen tube of *myo11c1-1*/*myo11c2-1* mutant than in the WT pollen tube. Lipid droplets velocities were measured for each frame by an automated algorithm. Speed measurements for each pollen tube were plotted as cumulative frequency distribution graphs (Figure 2b). Lipid droplets moved more slowly in *myo11c1-1*/*myo11c2-1* pollen tubes (green lines) than in WT pollen tubes (red lines) (Figure 2b). The average speed of LDs in the WT pollen tube was 0.22 ± 0.02 µm s^−1^ (Figure 2, Video S8). And the mobility of LDs in *myo11-2* (Video S9), *myo11a1-1*/*myo11a2-1* (Video S10), *myo11b1-1* (Video S11), *myo11c1-1* (Video S12), and *myo11c2-1* (Video S13) mutants pollen tubes were comparable to that of the WT pollen tube (Figure 2). Therefore, the trafficking of LDs in these mutants was very similar to that of the WT. Only in the double *myo11c1-1*/*myo11c2-1* mutant, the LDs’ trafficking significantly slowed down (0.16 ± 0.01 µm s^−1^) (Figure 2). Different from the latrunculin B, SMIFH2, and cytochalasin D treatments, in which the LDs’ wobble, the movement of LDs in the *myo11c1-1*/*myo11c2-1* mutant was only slower but did not wobble (Video S14). The results indicate that myosins XI are also involved in LDs’ trafficking in pollen tubes.

## 3. Discussion

Previous research on the mobility of LDs mainly focused on the seeds and vegetative tissues [11,38]. In animal systems, LDs’ trafficking is microtubule-dependent and is motorized by kinesins and dyneins [39,40]. The trafficking of LDs in plants has recently been studied in transgenic Arabidopsis, in which directional transport of LDs to the plasmodesmata at the leaf base involves the actomyosin system [11]. However, LDs in pollen tubes are far less investigated and it remained unknown what drive LDs movement in pollen tubes. In this study, the transport of LDs was not influenced by treatment with brefeldin A (Figure 1, Video S2), a vesicle formation inhibitor, indicating that vesicle formation is not involved in the movement of LDs. In tobacco pollen tubes, mitochondria move rapidly on actin but slowly on microtubules [41]. Here, we showed that the treatment of the microtubule depolymerizing drug oryzalin did not affect LDs’ movement and only exhibited a slight effect on the movement velocity of LDs (Figure 1, Video S3). In mammalian cells, microtubules mediate the assembly of F-actin [42,43]. In plant cells, actin polymerization may also initiate at microtubules [44]. The movement of LDs in pollen tubes does not directly rely on microtubules but microtubules might interfere with the actin system [41]. This could explain the weak effect of oryzalin on LDs’ trafficking (Figure 1). 

Pharmacological treatments reveal that the movement of LDs in pollen tubes was significantly reduced by the BDM, latrunculin B, SMIFH2, and cytochalasin D treatments, in which only residual wobbling of LDs was observed (Figure 1, Videos S4–S7). The results support that LDs’ trafficking in pollen tubes is driven by the actin system. Our data clearly proves that F-actin is pivotal for LDs’ movement in pollen tubes. The transport of LDs also relies on myosin [11]. While treatment with 10 mM or 20 mM BDM for 15 min did not reduce the mean speed of the movement of the LDs (data not shown), the transport of LDs was affected by treatment with 50 mM BDM for 15 min (Figure 1, Video S4). High concentration of BDM was required for inhibiting plant myosins, for example, 50 mM BDM, but not 10 mM was required to significantly inhibit cytoplasmic streaming and myosin mobility of *Chara coralline* [45]. 

Myosin XI was shown to be required for organelle movement, including mitochondria, peroxisomes, and Golgi bodies, in tobacco leaves [46] and in root hair [32]. Arabidopsis has 13 class XI myosins and seven of 13 myosin XI genes expressed in pollen: XI-A (Myo11A1), XI-B (Myo11B1), XI-C (Myo11C1), XI-D (Myo11A2), XI-E (Myo11C2), and XI-J. In *Arabidopsis thaliana*, *myo11c1-1*/*myo11c2-1* double mutants had defects in pollen tubes growth [47], but single mutants of *myo11c1-1* and *myo11c2-1* grow normally, and hence they may have redundant function in Arabidopsis pollen tubes [31]. In this study, relative to the LDs’ movement in WT pollen tubes, the LDs’ movement in *myo11-2*, *myo11a1-1*/*myo11a2-1*, *myo11b1-1*, *myo11c1-1*, and *myo11c2-1* mutants pollen tubes did not change significantly (Figure 2), and the movement of LDs’ trafficking was very similar to that of the WT (Videos S8–S13). Strikingly, LDs’ trafficking in the *myo11c1-1*/*myo11c2-1* double mutant slowed significantly (Figure 2) (Video S14), suggesting Myo11C1 and Myo11C2 are involved in LDs’ movement in pollen tubes. It has been reported that the movements of Golgi stack and peroxisome are strikingly reduced in *myo11c1*/*myo11c2* mutant in pollen tubes too [31]. Pollen tube growth is a polarized growth process that involves the functional coordination of multiple organelles. Together, myo11C1 and Myo11C2 are key myosins responsible for movement of organelles, including LDs, in Arabidopsis pollen.

The reason of having 13 class XI myosin genes in Arabidopsis genome implies that they have a division of labor (e.g., different cargos or interactors), while some of them may share certain degree of functional redundancy, such as Myo11C1 and Myo11C2, in Arabidopsis pollen. Different tissues may express different sets of myosins and their importance are different. For example, Myo11K has been shown to be associated with ER and is important for ER movement in leaf epidermal cells [26]. However, Myo11K is not expressed in the pollen tube [30]. This may imply that ER dynamic in pollen tubes is very different from that of the other cell types. In an earlier study, myosin XI-K and myosin XI-2 were shown to be involved in the movement of Golgi, peroxisomes, and mitochondria in root hair [32]. but a later study using YFP-tag myosin XI-K showed that while myosin XI-K colocalizes with actin, it mainly associated with endomembrane vesicles trafficking along F-actin rather than large organelles like the Golgi [48]. As XI-K is not expressed in Arabidopsis pollen [30], pollen tubes need to use other myosin XI isoforms, such as Myo11C1 and Myo11C2, for organelle trafficking. 

In summary, our results showed that LDs in Arabidopsis pollen tubes are trafficked by the actomyosin system and Myo11C1 and Myo11C2 are the major class XI myosins for LDs’ movement in Arabidopsis pollen tubes. Finally, it would be of great interest to examine the mechanism of how F-actin is involved in the LDs’ movement in pollen tubes. The connection between myosin-driven LDs’ movement, organelle movements and pollen tube growth are worth exploring in depth.

## 4. Materials and Methods

### 4.1. Plant Materials and Growth Conditions

Seeds of *Arabidopsis thaliana* var. Columbia (Col-0) and T-DNA mutants were surface sterilized. T-DNA lines (Table S1) were obtained from the Arabidopsis Biological Resource Center or from the authors [31]. Seeds were then plated onto half-strength Murashige-Skoog (MS) solid medium supplemented with 1% (*w*/*v*) sucrose. Seeds were cold stratified at 4 °C for two days to break the dormancy before being transferred to standard growth conditions at 22 °C in a 16-h-light/8-h-dark cycle. Ten-day-old seedlings were transferred to small pots (7 cm length × 7 cm width × 8 height) containing nutritional soil (Jiffy, The Netherlands) and then put in a growth chamber.

### 4.2. Pollen Germination

Pollen germination was performed as previously described with minor modifications [49]. Freshly open Arabidopsis flowers in 6–9 weeks old wild types or T-DNA mutants were harvested and mature pollen was germinated on the surface of solid pollen germination medium (1 mM CaCl_2_, 1 mM MgSO_4_, 1 mM Ca(NO_3_)_2_, 0.01 % (*w*/*v*) H_3_BO_3_, 18% (*w*/*v*) sucrose, and 0.8% (*w*/*v*) agarose, with the pH adjusted to 7.2–7.5 with KOH) in a Petri dish and incubated in a dark water bath for 3 h at 28 °C.

### 4.3. Nile Red Staining and Pharmacological Treatments

After pollen germination for 3 h, LDs in pollen tubes were stained with neutral lipid stain Nile Red (0.3 µg mL^−1^) for 15 min before microscopic observation. For pharmacological treatments, pollen germinated for 3 h was submerged in liquid pollen germination medium supplemented with 50 mM 2,3-butanedione monoxime (BDM) (Sigma, Livonia, MI, USA), 10 µM oryzalin (Merck, Kenilworth, NJ, USA), 50 µM brefeldin A (Sigma, Livonia, MI, USA), 40 µM cytochalasin D (Sigma, Livonia, MI, USA), 100 µM SMIFH2 (Sigma, Livonia, MI, USA), and 3 nM latrunculin B (Calbiochem, San Diego, CA, USA) for 15 min. The drugs and the Nile Red stain were washed away before confocal observation.

### 4.4. Microscope Images

LDs’ movement was observed using a Perkin Elmer Ultraview VOX Spinning Disc Confocal Microscope with a 100 × oil immersion lens equipped with a Hamamatsu C9100-23B EMCCD camera. LDs stained with Nile Red was excited by a 561 nm argon laser, and emission was collected between 580 and 700 nm. Time-lapse images were recorded in 2 s time intervals for 5 min. Nile Red has an emission peak at 635 nm, so there is no or very little emission in the green channel. The fluorescence signal of LDs in pollen tubes was recorded using the red emission filter at both t = 0 s and t = 6 s, and we assigned a pseudo-color (green) at t = 6 s so that the signals collected at 0s and 6s can be compared to each other.

### 4.5. Tracking LDs’ Movement with TrackMate

To track the movement of lipid droplets, live imaging data were analyzed by TrackMate in ImageJ 1.53 software (https://imagej.nih.gov/ij/, accessed on 28 September 2021) [50]. In detail, to track the LDs’ movement in pollen tubes, the pixel sizes, and time intervals (2 s) will be input in the tracking panel after the trackmate plugin was loaded. To filter out unreliable spots, multiple filters including quality, mean intensity, and X, Y with the quality filter were applied the most. The results of tracking were exported in an XML format file and the data was further analyzed by Rstudio (https://www.rstudio.com, accessed on 27 December 2021) [51].

### 4.6. Data Analysis

One-way ANOVA followed by Dunnett’s multiple comparisons test was performed by using GraphPad Prism Version 9 (www.graphpad.com, accessed on 7 June 2022). Sample normality was checked by the Q-Q (quantile-quantile) plot [52] and sample normality can reasonably be assumed as all data points are close to the 45° line (Figure S1). 

### 4.7. Accession Numbers

Sequence data used in this study can be found in the Arabidopsis Information Resource (https://www.arabidopsis.org, accessed on 22 May 2023) under the following accession numbers: Myo11A1 (At1g04600), Myo11A2 (At2g33240), Myo11B1 (At1g04160), Myo11C1 (AT1G08730), Myo11C2 (AT1G54560), and MYA11-2 (AT5G43900).

## Data Availability

All data are available in the main text or the supplementary materials. Materials are available from the corresponding authors upon request.

## Supplementary Materials

The following supporting information can be downloaded at https://www.mdpi.com/article/10.3390/plants12132489/s1, Figure S1: Sample normality analysis by Q-Q plot. Table S1: List of T-DNA lines used in this study; Video S1: LDs’ movement in WT pollen tubes (control); Video S2: LDs’ movement in WT pollen tubes treated with Brefeldin A for 15 min; Video S3: LDs’ movement in WT pollen tubes treated with Oryzalin for 15 min; Video S4: LDs’ movement in WT pollen tubes treated with BDM for 15 min; Video S5: LDs’ movement in WT pollen tubes treated with Latrunculin B for 15 min; Video S6: LDs’ movement in WT pollen tubes treated with SMIFH2 for 15 min; Video S7: LDs’ movement in WT pollen tubes treated with Cytochalasin D for 15 min; Video S8: LDs’ movement in WT pollen tubes (control); Video S9: LDs’ movement in *myo11-2* pollen tubes; Video S10: LDs’ movement in *myo11a1-1*/*myo11a2-1* pollen tubes; Video S11: LDs’ movement in *myo11b1-1* pollen tubes; Video S12: LDs’ movement in *myo11c1-1* pollen tubes; Video S13: LDs’ movement in *myo11c2-1* pollen tubes; Video S14: LDs’ movement in *myo11c1-1*/*myo11c2-1* pollen tubes. In all supplementary videos, pollen tubes were stained with Nile Red. Images were taken every 2 s for 5 min. The frame rate of each video was 1 frame per second. Scale bars = 10 µm
