# Supplementary material for: Movement of Lipid Droplets in the Arabidopsis Pollen Tube Is Dependent on the Actomyosin System
Source: Plants (Basel). 2023 Jun 29;12(13):2489. doi: 10.3390/plants12132489 (PMC10346980; doi:10.3390/plants12132489)
Supplement: Supplementary file 1 [file plants-12-02489-s001.zip › Figure S1. Sample normality analysis by Q-Q plot.pdf]

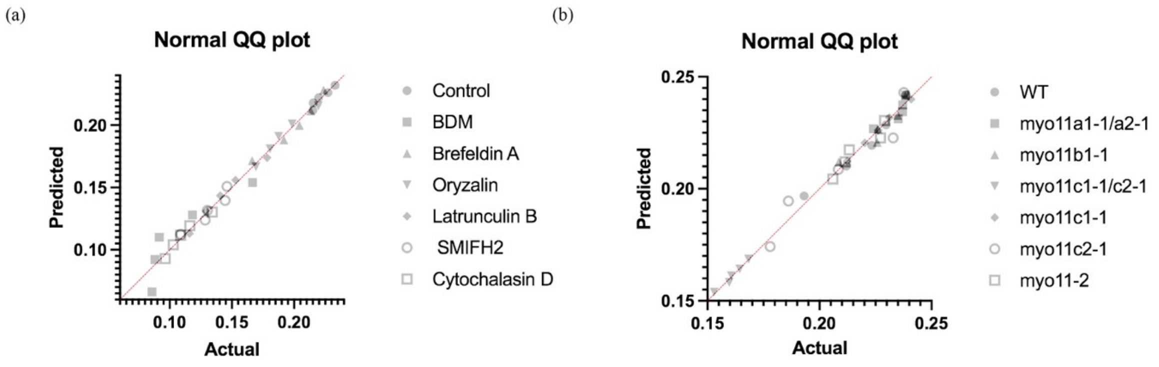

Figure S1. Sample normality analysis by Q-Q plot. (a) Normal probability plots for the mean velocity of LDs movements in pollen tubes treated with oryzalin, brefeldin A, cytochalasin D, SMIFH2, latrunculin B and 2,3-butanedione monoxime (BDM). (b) Normal probability plots for the mean velocity of LDs movements in pollen tubes of WT and *myo11a1-1*/*myo11a2-1*, *myo11b1-1*, *myo11c1-1*/*myo11c2-1*, *myo11-2*, *myo11c1-1*, *myo11c2-1* mutants.
