# Supplementary material for: Movement of Lipid Droplets in the Arabidopsis Pollen Tube Is Dependent on the Actomyosin System
Source: Plants (Basel). 2023 Jun 29;12(13):2489. doi: 10.3390/plants12132489 (PMC10346980; doi:10.3390/plants12132489)
Supplement: Supplementary file 1 [file plants-12-02489-s001.zip › Table S1. List of T-DNA lines used in this study.pdf]

Table S1. List of T-DNA lines used in this study.

Allele	Gene locus	TAIR accession	Insertion site
myo11a1-1/myo11a2-1	At1g04600/At2g33240	SALK_086989/ SAIL_607_G06	Intron/Exon
myo11b1-1	At1g04160	SALK_113062	Exon
myo11c1-1/myo11c2-1	At1g08730/At1g54560	SALK_129231C/ SALK_072023	Exon/Exon
myo11c1-1	At1g08730	SALK_129231C	Exon
myo11c2-1	At1g54560	SALK_089338C	Exon
myo11-2 (mya2)	At5g43900	SALK_055785C	Exon
